# Ideas and ideation in geographical political economy

**DOI:** 10.1177/03091325251326790

**Published:** 2025-03-18

**Authors:** Jamie Peck, Chris Meulbroek, Rachel Phillips

**Affiliations:** Department of Geography, 8166 UBC, Vancouver, BC, Canada

**Keywords:** Economic geography, geographical political economy, historical institutionalism, ideas, ideation, science studies, methodology

## Abstract

Departing from an engagement with the “ideas school,” a case is constructed for (distinctively) geographical political economies of ideation, first on their own terms and then in dialogue with three methodologically generative monographs. The power of economic ideas, the paper argues, is not located on the supply-side alone, in original texts, essentialized capacities or intrinsic qualities. These powers are realized situationally and relationally, through conjuncturally mediated interactions and translations. Since economic ideas are associated with powers, properties, and potentials that are contextual and geohistorically specific, rather than universal, there is a distinctive mandate for geographical political economies of ideation.

## I Introduction: (Where) do ideas matter?

The proposition that “ideas matter” is no longer an especially contentious one in the critical social sciences (Béland and Cox, 2010; Rueschemeyer, 2006), although *how* ideas matter, why, when, and for whom remain far from settled questions (Mehta, 2010; Swinkels, 2020). Since the inauguration of “the new sociology of ideas” (Block and Somers, 2018; Camic and Gross, 2001), an interdisciplinary repertoire of ideational approaches to political-economic explanation has been developed, spanning political science, historical institutionalism, economic sociology, (intellectual) history, and science studies. Notably, the “rediscovery of ideas” in comparative political economy (Blyth, 1997: 229; Jacobsen, 1995) has evolved into a loosely aligned “ideas school,” credited with a (trans)formative influence on an array of institutionalist research programs (see Béland and Cox, 2010; Hay, 2008; Kamkhaji and Radaelli, 2022). Complementing these efforts, new histories and sociologies of economic thought, policymaking, and practice have reached into state institutions and out into the vernacular worlds of consulting, think tanks, and the circuits of so-called thought leadership (see Appelbaum, 2019; Berman, 2022; Drezner, 2017; Liu, 2022; Mazzucato and Collington, 2023; Medvetz, 2012; Tsang and Cheung, 2024), punctuated by significant interventions at the nexus of intellectual history and heterodox political economy (see Mirowski and Plehwe, 2009; Mulder, 2023; Slobodian, 2018b). And not least, mostly cutting its own path, an influential research program in science and technology studies has been interrogating the diverse practices and performative effects of economic theories, models, and discourses (see Callon, 2007; MacKenzie et al., 2007; Weintraub, 2020).

Geographical political economists have registered these developments, but have yet to devote sustained or systematic attention to processes of ideation, or to the travels and transformative effects of economic ideas—although some notable exceptions attest to the more general rule. The latter includes adaptations of science-studies approaches in economic geography (see Barnes, 2001, 2004; Berndt, 2015; Christophers, 2013, 2014; Lave et al., 2010), but there have also been examinations of the spatiality of economic discourse and expertise (Gibson-Graham, 1996; Mallin and Sidaway, 2024; Massey, 2013; Meulbroek 2022; Meulbroek et al., 2023; Werner et al., 2014), and excursions into the (historical) geography of economic ideas (see Christophers, 2013; Mann, 2017; Peck, 2010; Peet, 2007; Sheppard, 2005); furthermore, there is an emerging current of ideational analysis in evolutionary economic geography (see Benner, 2024; Calignano and Nilsen, 2024).^1^ However, these contributions have yet to exceed the sum of their parts, prompting little in the way of programmatic development, methodological reflection or horizontal conversation across the field as a whole. Meanwhile, the most authoritative statement of the remit and rationale of geographical political economy, extending to some nineteen far-reaching propositions, does not explicitly identify a place for the role of ideas or the analysis of ideation—save for an acknowledgment of the discursive construction of economic relations, attributed supplementally to “the domain of cultural theory” (Sheppard, 2018: 169).

Consistent with the heterodox and ecumenical theory-culture of geographical political economy (cf. Bok, 2019; Peck, 2023; Pike et al., 2009; Sheppard, 2011, 2018), the paper asks what it might mean for this capacious and evolving project to specify an explicit role (and indeed place) for ideas and ideation, as something exceeding supplement or sideline. We take this step while noting the abundance of invitations to geographical engagement issuing from the broad field of heterodox political economy, in evocative concepts like thought collectives, expert ecologies, and epistemic communities (Cross, 2013; Farrell and Quiggin, 2017; Hirschman and Berman, 2014; Mirowski and Plehwe, 2009; Seabrook and Wigan, 2018); in the deconstruction of globalization projects, traveling models, and spatial imaginaries (Ban, 2016; Campbell, 2004; Slobodian, 2018b, 2023); in accounts of the role of ideas across cycles of crisis, failure, institutional transformation and consolidation (Best, 2020; Blyth, 2013a; Campbell, 1998; Hall, 2013); and in the demonstration effect of richly contextualized and situated modes of analysis and explication (Bockman and Eyal, 2002; Dezalay and Garth, 2002; Jabko, 2019; Rueschemeyer, 2006).

Yet if heterodox political economy can be credited with addressing “how, when, where, and why ideas … matter” (Schmidt, 2008: 305), the corresponding array of “where” questions has yet to receive the same degree of analytical attention, beyond commonplace attribution to “national differences” (cf. Blyth, 2002; Campbell and Pedersen, 2014; Hall, 1989). Confronting these questions directly, the point of departure for the paper is a sympathetic assessment of the ideas-school research program. We then go on to ask what a *geographical* political economy of ideas, conceived in its own terms, might look like. Our first step is to identify and elaborate those ontological and epistemological commitments that geographical political economy can be said to share with ideas-school approaches, including a qualified embrace of constructivism, axiomatic concerns with institutionally embedded power relations, and predispositions toward contextualized and conjunctural modes of analysis. But if these affinities and overlapping orientations are methodologically suggestive, they are hardly definitive or sufficient. This is our rationale for extending the discussion through an engagement with three contrasting models of ideational analysis, each of which is constructively adjacent to (but not *of*) the ideas school, while also being notable for their creative and distinctive handling of the *spatiality* of ideas: from anthropology, Hannah Appel’s (2019) poststructuralist ethnography of the reproduction of “offshore” oil economies; from sociology, Marion Fourcade’s (2009) comparative study of disciplinary traditions and intellectual formations in economics; and from history, Quinn Slobodian’s (2018b) alternative history of neoliberal globalization. Together, these monographs illustrate some of the diverse ways of “doing” ideas, each with resonances and implications for geographical political economy.

Our overall purpose is to emphasize the variety of methodologically tractable ways in which ideas and ideation can be productively engaged in accordance with the ontological premises, epistemological practices, and programmatic aspirations of geographical political economy. Furthermore, in building from foundations in heterodox political economy (where, in practice, “geography” ≈ national differences), we seek to advance a case for problematizing the contextual specificity of ideation, conceived as a socio*spatial* process. Along the way, and partly for heuristic reasons, areas of common concern and constructive difference will be identified with the extant science-studies tradition in economic geography, with its more practiced rationales and routines. This is not to polarize these approaches, as mutually exclusive or irreconcilable, but to sharpen the sense of what might be distinctive about *geographical* political-economy approaches to ideation, while opening up avenues for methodological experimentation and interpretative debate. To this end, the paper concludes with a distillation of implications, lessons, and rules of thumb for geographical political economies of ideation, grounded in the principles of conjunctural analysis. With this destination in mind, we begin by examining the rise of the historical-institutionalist ideas school, before summarizing and synthesizing the key tenets of (geographical) political economy approaches to ideation.

## II Ideas and ideation

There has never been a shortage of “ideas about ideas” (Schmidt, 2008: 306), such that judgment calls become necessary even at the stage of basic definitions. Although some ideas are characteristically understood to be “diffuse,” commonsensical, tacit or otherwise implicit, institutionalist researchers in the ideas-school tradition have been predominantly concerned with organized, “cognitive ideas,” including concepts, theories, and “explicit ideas about how the social world works,” or *should* work (Rueschemeyer, 2006: 228; Campbell, 1998: 384, 398; cf. Diessner, 2023; Helgadóttir and Ban, 2021). In this vein, we begin with the ideas school in focusing on those collectively held *economic* ideas that are mobilized in socially meaningful ways and/or with political intent, (re)producing vocabularies of “the economic” and knowledges, while in the process positing pertinent relations and putative causes; naming relevant actors and institutions, contexts and connections; and prefiguring, framing or “formatting” social action.^2^ This provisional definition encompasses organized models, projects, and paradigms, recognizing that these are always and everywhere subject to translation, contextualization, recombination, contestation, and embedding in habitual routines, normative practices, and “folk” or common-sense formulations.

The role of ideas in social action and political change has been a perennial concern in the social sciences (Camic and Gross, 2001), but the rapprochement with economic ideas and ideational analysis in heterodox political economy really began with the rise of the historical-institutionalist research program in the 1980s and 1990s.^3^ Influenced by catholic readings of Weber and Marx, historical institutionalists seek to analyze sociopolitical change with reference both to materially rooted power relations and to ongoing processes of normalization, institutionalization, and social learning. In contrast to rational-choice theorists and more orthodox Marxists (who tend for quite different reasons to attribute policy actions to calculable, material interests), historical institutionalists seek to construct midlevel, intermediate paths between the microanalytical and the macrostructural. They do so by prioritizing the mediating roles of political parties, social movements, corporatist institutions, and governmental organizations in the historically discontinuous movement of (dominant) ideas, governing paradigms, and disputed policy knowledges, refining their arguments through extended, critical-case investigations, such as macroeconomic crises, the evolution of welfare regimes, the long arc of the Keynesian consensus, and transformations in multilateral governance (see Abdelal, 2009; Evans et al., 1985; Hall, 1989; Rueschemeyer and Skocpol, 1996; Steinmo et al., 1992; Strange, 1988). A thread running through this work is that ideas are more than merely superstructural, but under certain circumstances assume socially consequential and causally significant roles in political-economic change.

Peter Hall’s (1993) seminal analysis of the crisis-enabled shift from Keynesian macroeconomics to Thatcherite monetarism is widely credited with initiating the ideas-school research program. His punctuated-equilibrium model posits a distinction between business-as-usual phases of economic statecraft, associated with relatively settled and broadly consensual ideas, and more episodic transitions *between* such normative regimes (or “paradigms”), when the very meaning of problems and solutions is contested, and when battles over ideas are joined, often in the throes of crisis. In this context, ideational paradigms are “internally coherent, composed of logically connected elements [originating] in fundamentally different theories of how an economy works and should work” (Carstensen and Matthijs, 2018: 432). Indexed to periods of political-economic crisis and transformational shifts in national economic governance, the notion of ideationally facilitated paradigm change became a staple for the ideas school, but also a locus of debate and contention (Berman, 2013; Best, 2020; Blyth 2013b; Carstensen and Matthijs, 2018; Hall, 2013), subsequently fostering working concepts like ideational robustness and resilience (see Carstensen et al., 2024; Schmidt and Thatcher, 2013).

If there was an early tendency to associate ideas with *deus ex machina* functions, as the exogenously given source of “‘road maps’ out of policy dilemmas” (for instance, in the recourse to “economic theories as explicit guides for reducing inflation [or] stimulating economic growth”), this would be supplemented by increasingly sociological investigations of the use of “symbols and other discursive schema [in order] to make these maps appealing, convincing, and legitimate” (Campbell, 1998: 381). In due course, “ideas, discourse, and processes of social construction [became] the core problematique” for the historical-institutionalist program (Blyth et al., 2016: 150; Schmidt, 2008), with its remit to explore “how entrenched, self-reinforcing policy paths tend to become stable and capable of resisting change even as environmental conditions continue to evolve and new and more efficient paths become available” (Carstensen et al., 2024: 3). Prevailing ideas have been shown to underwrite conditions of apparent normalcy (and “conventional wisdom”), just as competing or alternative ideas will under some circumstances undercut these selfsame conditions—especially in situations of crisis, which are often marked by (and sometimes defined by) elevated ideational contestation.

This orientation toward the causal and transformative capacity of ideas has opened up diagnostic pathways and foci for inquiry, leading into explorations of ambiguity, bricolage, recombination, and the creative repurposing of concepts, models, artifacts, and scripts in the ongoing work of socioinstitutional invention (Best, 2005; Carstensen, 2011; Wilder and Howlett, 2014). This concern with economic ideas as the grammar of political struggle and institutional (trans)formation, with the potential to exert decisive influence in moments of crisis and conflict, dynamized an historical-institutionalist research program otherwise prone to explanatory conservativism, with its default assumptions of incremental path-dependency under a relatively stable postwar world order (Abdelal et al., 2010; Ban, 2018; Berman, 2013; Farrell and Finnemore, 2009; Hay, 2008). The problematization of institutional discontinuity and disequilibrium called attention to the disruptive role of “new” economic ideas and novel ideational frames, particularly in the characterization of crises and competing strategies for their resolution, as well as during periods of governmental uncertainty, with ideational contestation intensifying around episodic challenges to faltering orthodoxies and failing paradigms (see Campbell, 1998; Hall, 1993; Hirschman and Berman, 2014).

The rise of the ideas school has been associated with the proliferation and deepening of *constructivist* approaches. Constructivists seek to explain how situations come to be as they are, rather than otherwise, and how they are shaped in relation to alternatives. Neither deterministic nor definitive, their accounts are concerned with cognitive worldviews and political beliefs, symbols and imaginaries, social norms and identities, together with contingent and contested processes of institutionalization. Institutions may structure and stabilize, but they are never permanent, static or complete. It follows that constructivist explanations are always partial and contestable, in fact categorically so. Properly understood as a pluralist enterprise, constructivism is animated by experimentation and ongoing debate between competing, contestable, and never-complete explanations. Constructivists differ around attributions of agency and causality, while demurring in principle from one-sided, deterministic or reductionist explanations. Some portray ideas as “causally powerful,” with a degree of independent influence over “the form and content of institutional change” (Blyth, 2002: 10), while others opt to explore contingent alignments and “elective affinities” between ideas, worldviews, politics, and institutions (Fourcade, 2009: 242). For the former, especially those sympathetic to the “paradigms” tradition in political science, a premium is placed on the attribution of the (sometimes overdetermined) power of ideas in steering political change. For the latter, such as those working in science studies or practice-oriented sociology, the influence of ideas is less direct and more mediated. Explanation is often couched in terms of the coevolution and congruence between ideas and fields like governmental policymaking, epistemic networks or the professions (see Berman, 2022; Dezalay and Garth, 2002, 2008; Fourcade, 2009; Sikkink, 1991). Here, ideas may not be the starting point or explanatory locus, but acquire meaning and salience through instituted practices.

Since its emergence as a subcultural trend within historical institutionalism, the ideas school morphed into the heterodox mainstream of critical international political economy and economic sociology. In the process, it has become a meeting point marked by the intersection of different theoretical approaches—neoGramscian, Bourdieusian, poststructuralist, and more—each engaging in their own way with the constructivist *problematique* of the relationship between the ideational and the social. Moving beyond these formative contributions, we next take three purposive steps toward conceptualizing the relationship between the ideational and the socio*spatial*. First, we develop the case for an approach to economic ideas in which place, context, and situation actually matter, above and beyond their background effects; second, we argue for a relational understanding of ideation as a power-laden, uneven sociospatial process; and third, we underscore the analytical and political contribution of conjunctural approaches to ideation, engaging and explaining its attendant practices and politics of ideation in concrete-complex and geohistorically specified terms. These theoretical considerations set the stage for an indicative examination of the variety of methodological practices that have the potential to complement, realize, and extend this approach.

### 1 Putting ideas in their place

The “ideas debate” has been marked by a persistent constellation of concerns with the non-deterministic interplay between potentially transformative ideas, contending forces and interests, and the moving terrains of political struggle and institutional change, as well as with recurring debates between more structural-materialist versus agent-centered or interactive approaches, and over the attribution and location of ideational agency. Across these positions, however, it is widely acknowledged that the causal capacity and intrinsic efficacy of ideas depend on context-specific circumstances (see Fourcade, 2009: 244; Rueschemeyer, 2006: 249). Since neither ideas nor the interests “behind” them can be said to speak for themselves, this means that ideas acquire meaning and salience only in context and conversation, through socioinstitutional mediation, even as they may subsequently appear to take on lives of their own (see Best, 2020; James, 2024; Widmaier, 2016). These particular articulations of processes and practices are not reducible to Humean rules of constant conjunction, since no universal or mechanical relationship between economic ideas and political-economic outcomes holds across cases and contexts, leading explanations in the direction of mutual conditioning, adaptative interaction, and complex relationality, but away from essentialism and foreclosure (see Blyth, 2002; Gofas and Hay, 2010; Hay, 2004, 2016).

Interpretative judgment calls made in this causally ambiguous and somewhat indeterminate terrain are precisely that. Ideas may prefigure, format or animate institutional and political projects, serving instrumental purposes for social actors and analysts alike—but they may also retrospectively *appear* to do so, as justifications, rationalizing devices or congruent narratives, sometimes invoked in real time, sometimes post hoc. (“Ideas that matter” may be called upon as the explanation of institutional change or socioeconomic transformation, or they may be *represented* as such, as “ideas that fit.”) Inescapably, the uneven traction and apparent periodicity of influential ideas must be, to some degree, in the eye of the beholder. “Visible,” significant or conspicuous ideas may be(come) recognized as such for circumstantial reasons (for instance, being advanced by charismatic ideational entrepreneurs, attending to legitimacy deficits or gaining recognition in urgent situations), as well as for putatively causal or explanatory reasons (for instance, as templates for transformative change). Ideas may even appear to “fail,” while nevertheless prompting reactions, adaptations, and innovations in the realm of practice, including the production of new devices, rules, rationalizations, and other such “concrete effects” (Best, 2020: 612). The nexus of circulating ideas and contextual efficacy is—and can only be—contingent and ultimately political. Few have expressed this more pithily than Milton Friedman (1982: xx), who in the post-Keynesian climate of the early Reagan years declared that, “Only a crisis—actual or perceived—produces real change … the actions that are taken [will] depend on the ideas that are lying around [once] the politically impossible becomes the politically inevitable.”

A defining characteristic of political economies of ideation, it follows, is their engagement with what might be called the “worlding of ideas,” including their construction, circulation, and reception. Beyond a concern with ideas in the abstract, this must encompass the places and fields of practice and translation, political mobilization and contestation, and the wider socioinstitutional and demand-side circumstances within which ideas, models, and interpretative frames acquire (or fail to acquire) salience. The reach of ideational analysis consequently encompasses what Best (2020) suggestively calls the “practical life of ideas,” tracing the movement and mutation of ideas not only as they travel “downstream” or radiate outward from some supposedly founding text, influential thinker or original source, but also to distill and diagnose their mediated effects “in solution,” in the intersubjective and hybrid worlds of practice, assimilation, mediation, translation, and institutionalization.^4^ This approach recognizes that while the so-called power of ideas may derive from their apparent potency or efficacy (as enabling imaginaries and frameworks for social action or as diagnostic and interpretative devices), it may also be a function of their flexibility, indeterminacy, and ambiguity, by virtue of their availability for “localized” customization, expedient repurposing and recontextualization.^5^ The power of ideas, in other words, is situational: “powerful” ideas may hail powerful actors or interests, or advance through institutional endorsement, but the powers that they possess (or acquire) only make sense in the (constitutive) context of agentic, social, and institutional relations. As Sheri Berman (2013: 229) has reflected:

the intrinsic attractiveness of any idea probably plays only a limited role in its chances of gaining political acceptance; instead, things such as its ability to attract clever and powerful champions, fitting into existing traditions and modes of thinking, and providing easily understood explanations and solutions to contemporary problems probably play significantly more important roles … Studying ideational change, in short, requires an examination of both the demand and the supply side of the equation––the creation of political spaces and the emergence of new ideas to fill them––and therefore an attention to *both* structural variables and impersonal forces, on the one hand, and case-specific factors and agency, on the other.

The practice of ideational analysis is consequently as much contextual as it is textual, as much socioinstitutional as semiotic. It must encompass the ways in which ideational powers are made, mobilized, and mediated. For Rueschemeyer (2006: 249, 227), in fact, there can be no such thing as a “general theory [of] how ideas matter,” nor “global answers” to the question of how, when, why, *and where* they matter; there can only be contextualized, conjunctural, and situated accounts. A telling illustration of this can be found in Block and Somers’ (2018: 155) analysis of “knowledge regimes,” or the “ideas, public narratives, and explanatory systems by which states and political cultures construct, transform, explain, and normalize market processes.” Their analysis of episodes of “ideational regime change” in the regulation of poverty (in nineteenth-century England and the United States after the civil-rights era) reveals how historically specific and purposeful mobilizations of moralizing and market-centric discourses come to “undermine, dislodge, and replace a previously dominant ideational regime” in the service of distinctive political projects (Block and Somers, 2018: 156, 177). If Block and Somers (2018: 155) court accusations of voluntarism or even idealism in their assertion that some “ideas have an independent influence on political outcomes,” their counter comes in the form of Polanyi’s embeddedness principle: for ideas to “work,” socially, politically, and institutionally, congruence and real-world traction must be established with (interpretations of) prevailing circumstances.^6^

Another illustration of the context-dependent nature of ideational analysis comes from the financial crisis of 2007-2013, and subsequent debates around the (perhaps unexpected) resilience of neoliberal ideas in the face of mounting deficits of credibility and legitimation (see Blyth, 2013b, 2016; Carstensen and Matthijs, 2018; Schmidt and Thatcher, 2013; cf. Peck et al., 2010). Blyth explains the post-crisis entrenchment of neoclassical and neoliberal formulations with reference to power struggles around ideas and institutions, meanings and representations, accounting for the outcome of “paradigm maintenance” in the following way:

[I]t is politics, not economics, and it is authority, not facts, that matter for both paradigm maintenance and change … [But it] is the incommensurate nature of rival claims that matters most of all … [T]he singular lesson of the recent crisis for the policy paradigms model is that the sociological can trump the scientific precisely because the locus authority did not shift *despite the facts*. Mere facts will (sometimes) not be allowed to get in the way of a good ideology … The struggle over [paradigmatic ideas] is a struggle over the meaning of anomalies, not their existence (Blyth, 2013b: 210-211, original emphasis).

There are, in other words, extrinsic and circumstantial dimensions to the vaunted power of ideas (see Bell, 2012; Berman, 2013; Campbell, 1998). The kind of ideas that achieve traction, those that “perform,” will do so under *conjuncturally specific circumstances*, circumstances that condition and contribute to the realization of powers that are not inherent, intrinsic or universal in effect. The charge for geographical political economists is consequently to put ideas in their place, tracing their mobilization, meanings, and diverse effects in situated circumstances. To do so means bridging between the discursive and the diachronic, text and context, conception and reception, accounting in the process for their iterative relations. And it also means transcending (or at least augmenting) the more conventional methodological and narrative registers of intellectual history, genealogy, and histories of economic thought, to embrace sociological, conjunctural, dialectical, and nonlinear modes of investigation that attend to questions of sociospatial construction, (situated) power struggles, and uneven processes of normalization, institutionalization, and crisis.

### 2 Ideas in relations

The articulation and advancement of political*-*economy approaches to ideation must entail more than a generic embrace of relationality, post-positivist methods or social constructivism; it is also more than an invitation to narrative enrichment by “adding ideas in.” It cannot be sufficient to call upon ideational rationalizations in an opportunistic or unprincipled fashion, merely to capitalize on situations where they can be plausibly aligned with or retrofitted to the play of events and the pattern of concrete outcomes, while otherwise overlooking their complex mediations and patterned effects (cf. Best, 2020; Bieler and Morton, 2018; Blyth, 1997). This raises the question of what obligations, commitments, and orientations attach *specifically* to political-economy approaches to ideation, mobilized with a geographical sensibility.

For political economists, “powerful” ideas are understood in relation to dominant interests, but not in a functionalist or “guaranteed” fashion. Economic ideas that acquire influence are probably best hypothesized as *a posteriori* functional for hegemonic social forces, tending to serve, extend, or (re)legitimize such interests in geohistorically particular ways. The species of ideas that challenge vested or dominant interests, on the other hand, frequently have to be formulated in such (oppositional, “alternative”) terms, predictably facing headwinds, if not explicit denigration, marginalization and sometimes suppression. In some cases, “alternative” ideas may be selectively appropriated, assimilated or co-opted in ways that enable powerful actors to “muddle through” periods of contestation (Carstensen, 2011: 158, after Lindblom, 1959). In contrast, as Trumpian discourse illustrates just as well as Chinese Communist party-speak, the forces assembled *behind* influential ideas can be such that these ideational formulations “need not bear much relationship to the reality they purportedly represent [since once they are] believed and acted upon, economic ideas have a tendency to become self-fulfilling prophecies” (Hay, 2008: 68; see also Baker, 2015; Baker and Underhill, 2015; Helgadóttir, 2022).

At the same time, ideational performativity is not a self-acting process, but instead mediates and proxies for power relations that can be considered “structural.” The political invocation of economic ideas—for example, in struggles over the distribution of resources and claims to legitimacy, which frequently entails the suturing together of putative causes to preferred responses, naming protagonists, scapegoats, winners and losers—calls attention to the stakes involved in identifying those actors, institutions, and interests assembled behind *what become* powerful ideas. It follows that the ideas themselves cannot be understood as (if) self-contained or self-acting “things” in themselves, as if discrete, essential, exogenous or antecedent. Recombinant in social and semiotic terms, ideas possess a compound, composite character (see Bockman and Eyal, 2002; Carstensen et al., 2024). They are deeply imbricated and mutually constituted with historically produced conjunctures, the instituted conditions and (internal) sociomaterial power relations of which are understood in dialectical terms (Jäger et al., 2016: 106). This informs a particular variety of constructivism, one practiced in a qualified and conditional manner, in conjunction and dialogue with the ongoing work of political-economic theorization—for instance, concerning modes and models of capitalist restructuring; dynamics of crisis formation, management, and (putative or revealed) resolution; mutual entanglements of social difference and uneven spatial development; projects and programs of regulatory transformation; and (trans)local economic imaginaries, visions, and schemes.

Such a “reconstructivist” orientation, which entails the ongoing work of critical theorizing within as well as across cases and contexts, marks one of the differences between political economy and science studies approaches to ideation. Ascribing to depth ontologies, political-economy approaches are concerned with the (re)organization of power blocs and dominant discourses, along with “the ideas and institutions that sustain them and the relationships between them” (Grayson and Little, 2017: 65; Hay, 2008). None of this need exclude (or minimize) the messier and more unruly worlds of networked and reassembled practice, the (flatter) terrain of characteristically occupied by science studies, where there is a predisposition to be somewhat distrustful of “larger explanatory schemes” (Law, 2017: 48). Science studies tends to favor self-consciously “smaller” concepts, detached or obliquely aligned with structurally patterned power relations, “theory, method, and the empirical [being] rolled together [as] all part of the same weave [which] cannot be teased apart” (Collier, 2012: 190; Law, 2017: 32; Law, 2004), often preferring to deconstruct, split from or “chip away” at more structural accounts rather than affirm or reconstitute what are portrayed as “stories about constant consistency and coherence” (Law, 2017: 48).

Yet rather than represent these differences between political economy and poststructural approaches as an unbridgeable ontological gulf, there is scope instead to reimagine this as a space of engagement between epistemological strategies—and between competing forms or projects of constructivist explanation. After all, political economy and science studies each have their own reasons for rejecting functionalism, reductionism, voluntarism, and economism, while also sharing overlapping concerns with the always-mediated and sometimes mercurial ways in which ideas come to matter or make a difference, never in isolation nor in circumstances separated from social context, practice, and politics. The defining science-studies position—that “the world is *not* open,” but neither it is foreclosed, since “[t]hings never *have* to be the way they are” (Law, 2017: 49, emphasis added)—can be read as a quintessentially constructivist stance that, in principle, few political economists would contest, even if they are generally inclined to give shorter shrift to agentic contingencies (see Gofas and Hay, 2010; Grayson and Little, 2017).

To sum up, political economy approaches to ideation are particularly concerned with the role of patterned and institutionalized power relations; the production, circulation and contestation of ideational projects and programs; and the (pre)conditions and strategic selectivities that shape the uneven adoption, deployment, and consequences of ideas within power-structured, geographically patterned, and contested sociopolitical fields (Hay, 2004; Sum and Jessop, 2013; Vadrot, 2019). Again, this is not to invite economism or reductionism, against which there must be ongoing checks. The analytical horizon must encompass, but also transcend, the domains of the intersubjective, the performative, and instituted orders of power/knowledge (see Ban, 2018; Bieler and Morton, 2018), acknowledging the conditions prevailing in different situations and terrains of struggle, but reaching towards more transformative visions, alternative imaginaries, and counter-hegemonic programs. Predicated on a relational conception of ideation, responses to this mandate resonate with Stuart Hall’s doubled-sided reading of articulation—as a discursive act, involving expressed attributions of meaning, and as an ontological condition, that of non-necessary connection or dialectical relation. To paraphrase Hall, ideational analysis is “a way of asking how [social ideas] do or do not become articulated, at specific conjunctures” (quoted in Grossberg, 1986: 53).

### 3 Situating ideas, conjunctural approaches

“Ideas,” for Stuart Hall, “only become effective if they do, in the end, *connect* with a particular constellation of social forces” (Hall, 1983: 82, original emphasis). This is not to insist on a narrow reading of the “ruling ideas” associated with dominant powers, but it is to problematize the contested *articulation* between ideas and the (specified) sociomaterial interests that are served by, and in various ways realized and refracted through, power-bearing ideas; it is to ask why it is that “a certain set of ideas, rooted within [specified] material relations, dominates at a particular point in time,” under some (but not other) conditions, and in particular places (Bieler and Morton, 2018: 75). This is to engage ideation as a worldly social process, articulated in persistent, but not predetermined ways with the patterned movement of social struggles, recurrent contradictions, and power relations. It is to recognize the protean, polysemic, and inescapably political nature of what Hall called *social ideas*, which “do not occur, in language or thought, in a single, isolated, way with their content and reference irremovably fixed” (Hall, 1983: 77).^7^ This translates into a position of measured skepticism concerning one-sided assertions of the intrinsic or supply-side power of ideas, or analyses otherwise detached or decoupled from the explicit theorization of geohistorical conditions and sociomaterial power relations.

There is a concern with the reproduction of hegemony here, but not in a rigid or monolithic sense. If hegemonies only exist in persistent states of reconstruction and contestation across moving terrains, situated struggles over socially articulated ideas are integral to this ongoing process. The ideas that achieve traction and influence will tend to exhibit (or establish) degrees of reciprocal congruence with the instituted and materially embedded ordering of power relations (see Béland, 2010; Bell, 2012; Bieler and Morton, 2018; Blyth, 2016; Parsons, 2016), against which social actors may mobilize, developing disruptive and alternative ideas in the effort to secure countervailing influence. Constructivists have elaborated theories and concepts to account for these movements. Carstensen and Schmidt (2016), for example, identify three axes of what they term “ideational power.” Persuasive power, or power *through* ideas, refers to the capacity to project and convert, to enroll others into ideational positions and perspectives on the basis of cognitive validity or normative value, frequently through the manipulation of narratives, symbols, stories, imaginaries, frames, models, and identities. Institutional power, or power *over* ideas, concerns the unevenly distributed capacities to control and dominate how ideas acquire meaning and salience, including the adoption/imposition of favored ideas, the exclusion of alternatives, and the outflanking of obstacles and sources of resistance. And hegemonic power, or power *in* ideas, refers to entrenched forms of common-sense, in which authoritative ideas become institutionalized, normalized, and structured in received thought. In moments of emergence and contestation, these may take the form of “social imaginaries” and prospective visions, allied to particular hegemonic projects (see Moulaert et al., 2016; Sum and Jessop, 2013).

Gathering the preceding arguments, we argue that geographical political economies of ideation should be wedded to explicitly contextualized, conjunctural modes of explanation, situated in worldly terms (see Clarke, 2015, 2023; Dixon et al., 2023; Jäger et al., 2016). Conjuncturalism, in this respect, is not just a perspective; it is also a position, and a geohistorically situated position at that. After all, it was the breakdown of Keynesianism, modernist projects of development, and the contested rise and uneven consolidation of neoliberalism, along with the proliferation of orthodox globalization discourses, that initially “animated a new generation of constructivist scholarship” in heterodox political economy (Abdelal, 2009: 66). It is no coincidence that the concepts, routines, and propositions associated with the ideas-school research program and its constructivist methods were advanced and debated in this *particular* geohistorical context, when a (social) premium was placed on the interrogation of (potentially) transformative ideas, their politicization, and their roles in radical socioinstitutional change (Campbell, 1998: 389).^8^ These circumstances were propitious, in retrospect, for the way in which they presented critical, real-time tests for emergent claims concerning policy paradigms, power-knowledge regimes, strategic selectivity, and the rupture and reconstruction of hegemonic projects. It is not that ideas *only* matter in periods or places of crisis, but they will tend in such situations to be more visible and voluble, sometimes more disruptive and decisive.

Developed in such circumstances, conjunctural approaches to ideation engage ideas in the context of historicized, grounded, and situated readings of political-economic conditions, articulated with (at least) medium-term movements in the uneven development and periodic rupture, crisis, and contestation of social formations. There is less of a concern with categorizing, defining, and bounding particular ideas in their own terms or for their own sake, but instead an insistent and searching (wider-angle) focus on their worldly encounters, purposeful translations, and contested invocations. Much of this will be engaged at the meso-analytical level, and indexed to historical, geographical, and institutional sources of conditioning and specificity, with an emphasis on the constitutive connections between the micro and the macro, the agentic and the structural, rather than privileging one at the expense of the other. This suggests approaches to conjunctural inquiry that gravitate towards politically contested sites and situations, interrogating problem spaces and arcs of crisis management with recourse to conceptual metaphors like regime, paradigm, and (social) formation (Clarke, 2023; Grossberg, 2019). The intention here is to grasp (and to ground) the macro as an extended scalar domain across which super-emergent and more-than-local properties are manifest, and where processes of interdependent, uneven, and combined development are registered. This also facilitates one of the objectives of conjunctural inquiry, to explore and problematize part-whole connections in relational and comparative terms, across a diversity of grounded and situated contexts (cf. Hart, 2018; McMichael, 2000; Meulbroek 2023; Peck, 2023, 2024).

## III Models and methods

The preceding discussion marshalled some of the distinctive ontological orientations and epistemological commitments associated with political-economy approaches to “spatialized” ideational analysis. For all of their methodological implications, however, these approaches are neither complete nor discrete. Ideational explanation is more a matter of different strategies of representation, the balance of arguments, and choices made in interpretation than it is about the imposition of binding methodological rules or the hammering out of definitive, once-and-for-all conclusions. Despite the well-known differences between science-studies treatments of “soft realities,” in which the ideational is “practice embedded [and] location dependent” (Law, 2017: 43, 40), and the priority that political economists typically place on the structuring role of institutionalized power relations, along with more hard-edged takes on the ideational, we have suggested that it is counterproductive to polarize these approaches, given the opportunities for debate and dialogue across the spaces in between. Indeed, there are reasons to value encounters between competing methodological approaches on the contestable terrain of constructivist explanation.

In the spirit of such a positive-sum approach to methodological diversity, we now turn to a focused discussion of three monographs—Hannah Appel’s *The Licit Life of Capitalism*, Marion Fourcade’s *Economists and Societies*, and Quinn Slobodian’s *Globalists*. Our criteria for selecting these among many potential candidates are threefold. First, they extend the registers of the ideational, each featuring substantive, contrasting engagements with the grounded spatiality of political-economic ideas in ways that are complementary to, but distinct from, the historical-institutionalist mainstream of the ideas school. Second, in their contributions, respectively, to poststructuralist anthropology, to comparative economic sociology, and to critical intellectual history, these monographs speak to different ways of “doing” ideational analysis, exemplifying different dimensions of what ought to be a diverse and open methodological repertoire. And third, by highlighting various silences, congruences, and touch points with extent or emergent methodological currents in economic geography, they are indicative of some of the different ways in which questions of space, place, and scale, or grounding, worlding, and contextualization, might be taken up in geographical political economies of ideation, beyond the methodological routines of the ideas school. In what follows, we engage each book on its own terms (and terrain), before briefly picking out some cross-cutting themes.

### 1 Ethnographic encounters: Ideas in the wild

The treatment of economic ideas in science studies and poststructuralist anthropology is notable for the attention afforded to heterogeneous and nonhuman forms of “micropractical” agency, and to the performance and translation of ideas through (actor) networks and ordering frames across spatially or relationally adjacent fields (see Ban, 2018; Bockman and Eyal, 2002; Callon, 1998). Beyond the pioneering line of work on the laboratory as a site of experimentation, after Callon (2007; Callon and Rabeharisoa, 2003), science-studies approaches are also notable for their encounters with ideas as they propagate through networks and manifest (variously) “in the wild.” In *The Licit Life of Capitalism*, Hannah Appel tackles the very stuff of political economy (the reproduction of capitalism, corporate extractivism, labor relations) with tools derived from theoretically informed ethnographic inquiry. This poststructuralist variant of ideational analysis is concerned with the concrete and experiential settings in which shared understandings emerge, how they are constructed through social relations “in place,” and how ideas are (dis)embedded, localized and delocalized through social networks (cf. Barnes, 2004; Shapin, 1998; Sheppard, 2005). Appel (2019: 23) works with and through “the complex entanglements, histories, and multiplicities of daily life” to inquire into how Equatorial Guinea’s oil economy comes to be “sublated into something called ‘global capitalism’,” the definition and received character of which are understood to be “made” (and locally remade) rather than fixed or given. The book is concerned with the production of a kind of normalcy (in some ways, normalized conditions of dysfunction and crisis), with projects of standardization and stabilization, and with the work that goes on to produce the *very idea* of capitalism in this situated setting, including its licit character and its “smooth” façade—despite ongoing corruption, racialization, insecurity, and maldevelopment.

*Licit Life* does not take abstract ideas as a point of departure. Instead, tracking “the economy” through everyday spaces, sites of reproduction, corporate enclosures, staged events, Appel takes the work of abstraction itself as an object of ethnographic inquiry. Economic theories like the resource curse become “found objects,” read here in performative terms, generating concrete and semiotic effects as they imperfectly enact the worlds that they purposefully disclose and purportedly describe (see Callon, 1998; MacKenzie et al., 2007; Miyazaki, 2013). Furthermore, received and constructed categories—including some of the most taken for granted, such as the “national economy”—are seen to flatten and format economic life into statistical representations, in this case (re)producing images of the oil industry as a benevolent engine of growth and development (see also Mitchell, 2002, 2005). Ideas *qua* ideas are among several depoliticizing technologies (such as contracts), that together accomplish these feats of distancing, abstraction, and (mis)representation, helping to make capitalism *appear to be* orderly, rule-governed, and “above” local entanglements. There is no taking for granted in Appel’s research practice; indeed, the taken-for-granted is itself rendered as a space and indeed object of inquiry:

There are at least two kinds of “not-noticing” during fieldwork. There are the things you don’t notice because you rarely come across them, and there are the things you don’t notice because you come across them so frequently (Appel, 2019: 137).

Notably, Appel (2019: 22) engages capitalism not as a pregiven operating environment, inherited institutional structure or what she frames as “context,” but as an ongoing “project,” and more specifically as a “constant construction project to be followed through research.” *Licit Life* explores a range of sites through which these capitalist projects are *made*—the concrete spaces of the offshore and the enclave, the legal framing of contracts, statistical constructions of the national economy, and the political ideal of transparency—each exemplifying what Appel calls the “as ifs” upon which capitalism itself depends: abstraction, decontextualization, and standardization. The corresponding ethnographic task is not simply to expose these as capitalist fantasies, or to depict the gulf between smooth representation and messy reality. Rather, the fantasy and the work of projection become analytical concerns, documented through thick descriptions of the everyday routines and habituated practices that render such normalized facets of economic life as legitimate, efficient, productive, and rational. Appel’s concern is with the downstream, demand-side and worldly “effects” of economic ideas, with their consequential “lives,” and with the myriad ways in which they are rendered licit. Economic ideas cannot be analyzed apart from the sociomaterial worlds through which they are enacted, since their significance lies with the work that they perform in projects of smoothing and overwriting the embedded and embodied frictions of capitalist development.

Explicit and recognizable economic ideas like resource-curse theory are encountered in *Licit Life* as artefacts in the field: Appel reads this “ubiquitous and traveling form of economic theory” for its productive effects in normalized, everyday understandings, in politics, and in future-oriented imaginaries. Following Callon (1998, 2007), these performative effects are shown to include the production of “distance” between the corporate capitalism and its sociomaterial consequences (see also Berndt and Boeckler, 2009; MacKenzie, 2005, 2006; MacKenzie and Millo, 2003). These distancing effects are “aspired to and fought for” through a variety of “bodily, affective, and technical practices,” including the mobilization of economic theory: oil company managers invoke resource-curse theory to distance themselves from local politics, while elites used the idea to apportion blame and responsibility for the country’s economic condition, pointing to conditions (naturally) associated with the resources themselves, rather than “at the feet of power” (Appel, 2019: 23, 221). The work of economic theory in this context is to posit a (politically convenient) connection between the properties of hydrocarbon and the pathologies of oil-exporting states, while “disappearing” the corporation. Oil duly emerges “*as if* untouched” by the corporate hands involved in schemes for its extraction (Appel, 2019: 213, original emphasis).

More than the exclusive preserve of professional economists and policymakers, economic ideas are shown to pervade the vernacular languages through which politicians, local elites, transnational experts, and corporate managers labor to sustain projects of capitalist development. The circulation of “ideas about lawlessness,” for example, becomes implicated in justifications for “modular” forms of extractivist capitalism, insulated and gated off from host communities. This is a quite different approach to that of following ideas to (or out from) “truth spots,” linking them to upstream spaces of knowledge production or back to sites of supposed origination. Instead, Appel tracks how ideas travel in socially mediated ways through a multiplicity of spaces and networks, many of them in the “mezzanine tiers” of economic life (Kintzi, 2021: 482). As an investigation into the (re)production of smoothness, there is some smoothness in this deftly handled ethnographic analysis too. There is less of an explicit concern with the construction of ideas themselves, with their sometimes-disruptive role in wider social struggles, or with contoured or variegated formations of capitalism, in its unevenly developed forms or in competing hegemonic projects. Instead, the concern with abstraction extends to capitalism itself, which is encountered in closely focused and grounded settings, but less often in wider angles.

Connecting with longstanding geographical debates over the production of space (cf. Goswami, 2004; Sheppard, 2018), *Licit Life* documents how oil companies actively reconstruct understandings of distance and connectivity as they negotiate contradictions between aspirations for the seamless and unimpeded global flow of capital and entanglements with local sites. This reconstruction takes place through practices of distancing and decontextualization, many of which are rendered material (e.g., the creation of spaces like the enclave and the offshore). Appel is primarily concerned with the downstream assimilation of ideas into everyday practices and received formulations. The ideas do not work all by themselves; they are purposefully put to work. So it is that the oil industry *uses* “anthropology, history, economics, and political science to efface the agency of transnational corporate capitalism” Appel (2019: 5) demonstrating that the (re)production of economic knowledges and ideational technologies can have real “bite,” not least “in the mouths of some of our world’s most powerful corporations.”

### 2 Comparative sociologies: Cultures of economics

Rather than assume that states act as “transmission belts” for underlying socioeconomic forces or sites for the realization of pregiven material interests (Berman, 2013: 218), the historical-institutionalist research program demonstrated how specific institutional structures affect the social organization of knowledge, pursuant to the plenary claim that policy-relevant ideas frequently shape the content, course, and consequences of state action. To this end, much of the early work on the diffusion of Keynesianism “across nations” grappled with the (potentially) causal relationships between the supply-side of policy-facing economic knowledge and the demand-side of institutional transformation (Hall, 1989; Skocpol and Finegold, 1982; Weir and Skocpol, 1985). Questions concerning *where* these varieties of economic knowledge were being produced and reproduced, as distinctive forms of expertise, tended to escape explicit analytical attention, however, beyond the necessary if insufficient recognition of “national differences.” Marion Fourcade’s (2009) *Economists and Societies* represents a significant and original intervention in this respect, as a systematic and rigorously comparative attempt to understand how and why it is that prevailing economic ideas and the cultures of academic economics differ from country to country over time—even under the sway of a tendentially monist and “universalistic” disciplinary culture (Economics, in its capitalized form), which itself tends to be dismissive of contextualized explanation. Fourcade’s (2009: 3) deeply historicized account depicts the institutionalized lives of a discipline that is not nearly as singular as its preferred (self) representations, demonstrating that from the start, while “economics arose everywhere … everywhere it was distinctive.”

*Economists and Societies* demonstrates that varieties of economic knowledge and expertise, linked to reservoirs of policy-relevant ideas, reflect geographically specific institutional orders. Fourcade refuses to posit a one-way causal relationship between ideas and institutional change, being less concerned than other institutionalists with the making and breaking of paradigms (cf. Blyth, 2002; Hall, 1993; McNamara, 1998), since ideas are seen to be produced within (and largely endogenous to) institutional domains. Instead, she explores the “elective affinities” between the theories-*cum*-practices of economics and the institutionalized character of academic and governmental orders, in other words, the nationally scaled and constituted “*coevolution* of politics, policy, and ideas as they are shaped by social and institutional structures” (Fourcade, 2009: 238, 242, emphasis added). Ideas are analyzed as the medium and mediated outcome of long-run patterns of institutional development around the (re)production of the discipline, the profession, and the policy-influencing work of economics across national “fields” of knowledge. The constructivist contribution here is to reveal how norms, conventions, and meanings are produced and ordered in different (geographical) settings, between (academic) discipline, (social) organization, and (political) practice.

Following Bourdieu (1975), Fourcade takes the discipline and profession of economics, with its internalized ecosystems of reward, hierarchy, and sense of mission, as the principal field through which ideas are produced, distributed, and governed. Within and across her three cases—the United States, Britain, and France—she analyzes varieties of economics as fields structured in relation to orders of learning (the positioning of economics within the higher education and scientific research system), administrative orders (the differential incorporation of economists into governmental organizations), and economic orders (the place of economic technologies in the system of economic relations). Her focus is less on the (competing) positions and theoretical ideas of prominent economists and more with the patterning of ideational cultures within nationally constituted orders, read as coexistent and coevolving styles of economics. Meticulous documentation and detailed description is combined with more boldly drawn distinctions, rendered in broad-brush terms: the United States is shown to possess a meritocratic class of “merchant professionals,” defined by deference to the market; the culture of economics in the UK is shaped by public-minded elites, oriented to establishment power centers; and consistent with the engineering ethos characteristic of French economics, technocratic knowledge is placed in the service of an administrative state.

*Economists and Societies* exemplifies a rigorously constructed cross-national research design, one that is relatively rare in geographical political economy but which represents something of a staple of economic sociology and historical institutionalism. Despite the inevitable stylization entailed in depictions of distinctive “national” traditions, this attention to geographically specific normalization and institutionalization is not to be confused with a default reliance on methodological nationalism. Fourcade’s carefully explicated methodological strategy—“critical organized comparison”—unpacks spatiotemporal variability down to the constitution of variables and the categories of comparison themselves, while also serving as the conceptual framing for the book.^9^ The very meanings attributed to “the economic,” including what it means to “be” an economist, are themselves matters for probing inquiry and cross-case comparison. Nevertheless, attachment to the national scale of analysis does imply certain trade-offs, privileging some connections, institutions, and relations while marginalizing others. One implication is that the realm of the international is relegated to secondary status (although see Dezalay and Garth, 2002, 2008; Fourcade, 2006), even as the three national cases are never simply portrayed as independent, self-contained units or simple equivalents. Recognizing these issues, Fourcade approaches her national cases as a mutually referential selection of hierarchically articulated and unevenly ordered fields. For example, in the early years of American economics, there was deference to the ostensibly superior training provided by German universities along with valuation of such “foreign linkages,” while the economics professions of Britain and France are shown to be subject to (uneven and incomplete forms of) “Americanization” after the middle of the 20^th^ Century (Fourcade, 2009: 65, 140, 163). Even in these crossover contexts, however, nationally scaled institutional processes are shown to imprint path-dependent effects on the wider trajectory of “global” economics.

As Mirowski (2009) observes, this focus on national comparisons cannot but underplay the changing positions of rival schools of economics *within* national borders (see also Henriksen et al., 2022). Some of the work that has followed in the wake of *Economists and Societies*, however, has taken up this problem. In *Think Tanks in America*, for example, Medvetz (2012) offers a field-analysis of these distinctively positioned and professionalized policy-knowledge organizations, theorized (also after Bourdieu) as interstitial domains constituted through overlapping relations with academia, government agencies, media, and private business, and engaged in ongoing struggles to balance political access and relevance, public credibility and visibility, and access to funding. In a parallel fashion, Berman’s (2022) *Thinking Like an Economist* documents the ascendancy of an increasingly pervasive “style” of (micro)economistic reasoning, based on marginalist cost-benefit analysis, within the armatures of the US federal government. She traces the migration of this economic style from the Vietnam-era rationalization of defense programming to the administration of social policy in the context of Johnson’s Great Society, prior to its more generalized diffusion through graduate-training programs in public administration and accepted forms of professional and budgetary practice.

In contrast to theoretically informed ethnography of economic knowledge presented in Appel’s *Licit Life*, where ideas are found almost exclusively in impure states of “solution” and everyday application, circulating by means of enabling techniques, accounting devices, and the labor of an heterogeneous assortment of actors, Fourcade’s sociology of knowledge approach concerns itself almost exclusively with the professionalized worlds and expert communities of economics, mobilizing the comparative method in the service of a revelatory analysis of contrasting institutional ecosystems and the geographically differentiated “relationship between ideas and institutions” (Campbell, 2010: 1608). Methodological differences aside, there is nevertheless a shared concern here with the embedded, embodied, and constitutively contextual lives of economic ideas, contra notions of exogeneity, a concern foreshadowed in Fourcade’s (2009: 229) observation that “[t]he economy as an object is a constantly moving target that cannot be separated from the projects, designs, and practices of economists—nor, for that matter, from the projects of accountants, financiers, planners, statisticians, lawyers, [and] consultants.”

### 3 Intellectual histories: Upscaling neoliberalism

Although some contemporaneous accounts of the ascendency of neoliberalism emphasized its distinctively ideational dimensions (see Desai, 1994; Gamble, 1988; Hall, 1983), these takes have since been enriched and deepened by a new generation of intellectual historiographies (see Jackson, 2022; Mulder, 2023; Phillips-Fein, 2019). Rejecting ideational originalism, as if ideas prefigure practice by cascading downstream from Mont Pèlerin, this more recent literature has documented the adaptability and generative heterogeneity of neoliberalism as a power-knowledge regime, the multiplicity of its origin stories, and the complex relations between theorists, “secondhand dealers in ideas,” policy entrepreneurs, and front-line political actors, as well as between germinal texts, instrumental translations, and institutional transformations. As Ben Jackson has observed:

Much of this work has moved from the inside out, focusing on reconstructing the history of thinkers, activists, politicians, businesspeople and organizations who have nurtured neoliberal ideas which then go on to play a role in policy or public discourse. But intellectual historians can also move from the outside in, evaluating the significance of neoliberal ideology in reshaping the [contemporary] political landscape … compared with—or in combination with—other distinct ideological and cultural trends (2022: 985).

Inside-out approaches start with thinkers and texts on the ideational supply side, the direction of explanatory travel being from “original” sources out (or down) to social situations, from texts to translations and transmutations, and from concepts to contexts. Moving in the opposite direction, outside-in approaches cut in from the demand side and the historicized present, distilling ideational currents from complex social situations. Conventional intellectual histories tend to favor the first approach: as forward narratives, they characteristically depart from seminal texts, prescient thinkers or formative debates, before venturing downstream from this elevated terrain to the marshy lowlands. In contrast, to recover or excavate ideas from the thickets of particular social institutions, geohistorical moments or the here-and-now is to work in the opposite (upstream) direction. Characteristically deconstructive, outside-in analyses are often more circumspect when naming definitive ideational sources (cf. Collier, 2011; Offner, 2019; Ong, 2006; Rodgers, 2011). Appel’s *Licit Life* likewise encounters ideas in the wild, not in anterior or “pure” form.

Beginning with its own version of an inside, but reaching far beyond, Quinn Slobodian’s *Globalists* strikes a balance between a “group biography” of neoliberalism’s (lesser known) Geneva school and a searching exploration of its designs on the (re)making of multilateral institutions, reconstructing a hitherto-neglected strand of “neoliberal thought *on its own terms*” (Slobodian, 2018b: 8, emphasis added). Slobodian advances an alternative political-economic history of the 20^th^ Century (the “neoliberal century”), proposing nothing less than a “new frame for the history of [the neoliberal] movement” (Tooze, 2018: 132). Here, the Geneva school’s slow-burning project of market-protecting constitutionalism takes shape as a dialectical response not to the limits and crises of 1970s Keynesianism, as now-conventional accounts have it, but instead to a much earlier era of historical dislocations: the First World War and the end of empire. The geohistorical arc of neoliberalization itself consequently rendered anew, beginning as a reaction against popular enfranchisement and democratization (insulating markets and property from politicization), before evolving more programmatically in opposition to economic managerialism (and the interventionist conceit that national and global economies are knowable and therefore governable).

Combining methodological discipline with explanatory ambition, Slobodian (2018b: 19, 24) concentrates on a “relatively small number of individuals,” a cabal of Geneva-adjacent prime movers, plus an array of second-tier allies and enablers, in the service of a “fairly constrained story” with quite sweeping implications. Anchored in central Europe, but reaching far and wide, this sociologically inflected history involves a close and critical engagement with neoliberal thinkers, theories, and imaginaries. Slobodian’s reading unfolds outward from ideas and texts to the more nebulous territory of (shared) worldviews and aspirational projections, grounding these in the concrete geographies of the Geneva school but radiating out through an overlapping series of world-historical events, from interwar Vienna to apartheid South Africa, from the Suez crisis and the origins of European integration to the genesis of the World Trade Organization. Although light on methodological reflexivity, there is a clear affinity with “thought collective” sociologies, with their emphasis on the intersubjectively processed reproduction of transformative ideas (Mirowski and Plehwe, 2009).^10^ Accordingly, the book speaks to audiences in the critical social sciences, engaging squarely with (now mature) debates around the political economy of neoliberalization, if for the most part demurring from explicit theorizing (see Sparke, 2019), while amending extant histories and geographies of neoliberalism (see Stafford, 2019; Streeck, 2019; Tooze, 2018).

Consistent with inside-out approaches to the study of neoliberalism's history, the book’s epistemological ambit is centrifugal, radiating out from what initially resembles “a sect, slowly turning into a church, of Gramscian ‘organic intellectuals’” (Streeck, 2019: 839).^11^ Even as intellectual networks are knowingly taken to be a starting point, rather than “a Rosetta Stone to understand neoliberalism as a whole (Slobodian, 2018a: 2), comprehensively inoculating this approach from charges of confirmation bias is an enduring challenge, be this through synecdoche or the tendency for “projected” readings to order the world in self-referential terms. Working from the inside out means encountering the messier, downstream worlds of social struggle, quotidian practice, and ongoing translation only secondarily and sequentially. Outside-in approaches, in contrast, begin in the realm of lived experience or political practice, prior to teasing out strains and subcultures of neoliberal thought among their others.^12^ Slobodian’s investigations reach the thresholds of institutional and grounded political struggles, but do not venture within. Crucially, the latter is where claims as to the actual *influence—*as distinct from affinity or congruence—of neoliberal ideas are to be adjudicated, in theoretical as well as empirical terms. Slobodian (2018b: 119) acknowledges that the institutional architecture of the postwar order was “designed with little or no input from the neoliberals themselves,” even as its concretely realized forms frequently rhyme with Geneva tunes.

A recurrent (if somewhat latecoming) theme in critical investigations of the historical arc and uneven geographies of neoliberalism has been “the central role [that] ideas played” (Centeno and Cohen, 2012: 317; Mirowski and Plehwe, 2009; Mulder, 2023). A decisive contribution of *Globalists* has been to augment and reorientate received (critical) understandings of the ideas “behind” neoliberalism, not least to extend their scope. To remap their contours, and to upscale their dialectical reach, from the global imaginaries born out of the ruins of empire through to projected schemes for multilateral governance. A function of this epochal scope is that an upward-scaled historical narrative tends to take precedence over “lateral” questions of uneven development, localized governance, and relational interdependence. The book does not surrender to simple diffusionism, but the traces of methodological centrifugalism inevitably remain in an argument indexed to “Geneva thought.” To construct an ideational narrative from the inside out is to confer a germinal measure of explanatory centricity, since it is ideas that both define the source and illuminate the path. This said, the Central Europe of the interwar and midcentury years is some distance, temporally and spatially, from what are more conventionally invoked as the “heartlands” of post-1970s neoliberalization (see Jessop, 2016; Slobodian and Plehwe, 2022).

It is axiomatic for Slobodian (2018b: 34) that “[n]eoliberal thinkers arrived at their ideas in response to the world they saw around them,” in a situational sense, as a matter of worldview, and in terms of future-oriented imaginaries. Here, a pivotal inflection point is an ideational turn engineered by Central European neoliberals, away from projects of statistical representation, the measurement of business cycles, and other schemes of “world observation,” in favor of an almost metaphysical conception of the global economy as “sublime,” ineffable, and “beyond capture” (Slobodian, 2018b: 57, 87). This lends itself to an alternative imaginary, “thinking in orders,” which for Slobodian sits at the heart of ordoliberalism. After Carl Schmitt, this was (re)conceived as a “doubled” world, divided between the “dominium” of market dynamics and the “imperium” of territorially demarcated nation states, yielding the advocacy of constitutional measures to restrain and contain politicization while protecting market rule. Slobodian’s is a probing analysis of this meta-constructivist program, delivering “not only a genealogy but a feel for [its] institutionalizing imaginary [and] unfolding institutional facticity” (Weiner, 2023: 203). In so doing, he ventures further than most from the interior of one of neoliberalism’s lesser-known yet projects of germination to the outer reaches of its influence, if less than all of the way into its institutional lifeworlds and social contestations.

### 4 Reading for ideation: Extending methodologies

Our reading of the preceding three monographs has had to be both limited and somewhat telegraphic. The specific purpose here has been to deploy selective readings of a small but diverse sample of exemplary texts as a means to extend the field of methodological vision-*cum*-practice, beyond the routines of the ideas school and in anticipation of emergent pathways for geographical political economy. The three books each have their own ways of “locating” and then problematizing ideas, according different meanings and explanatory weights to processes and practices of ideation. In Hannah Appel’s *Licit Life*, ideas are encountered in their vernacular, grounded, and artefactual forms, sedimented in (often tacit) social practice, courtesy of a method of ethnographic casing that ranges fluidly from the scales of the local and national to the global. This treatment of ideas in solution, rather than in pre-social abstraction, resonates with methodological dispositions in key strands of geographical political economy, by staying close to practices in their diverse, localized, and networked forms, by attending to the performative qualities of models, visionary schemes, and formatting devices, and by tracing distributed forms of (more-than) human agency. While she does not begin with ideas, Appel is keenly attentive to the work that they do, in their everyday forms, with how they fuse with received practices and understandings, and not least, with the ways in which these ongoing projects sustain the idea of capitalism itself.

If Appel’s *Licit Life* models a methodologically disruptive treatment of the taken-for-granted context occupied by corporate capitalism, Marion Fourcade’s *Economists and Societies* is disruptive in a quite different way, as a geographically staged, critical counter to the performative monoculture that is economics, in its academic, “applied,” professional, and political forms. If crossover opportunities abound in the methodological trading zone between geographical political economy and Appel’s style of poststructuralist ethnography, this is perhaps less obviously true of Fourcade’s comparative approach to the sociology of economic knowledge. Fourcade’s rigorous and reflexive research design, however, is surely also worthy of emulation in geographical political economy, where an ingrained aversion to methodological nationalism has permitted the neglect of this undeniably consequential scale, as well as missed opportunities to connect with international political economy, economic sociology, heterodox political science, and related institutionalisms. Fourcarde’s original blend of field theorizing and “critical organized comparison” calls attention to some of the roads less traveled in political-economic geography, where methodological experimentation on this front continues to lag. Finally, *Economists and Societies* is also distinguished by its deft combination of long-range institutional analysis, archival inquiry, and expert interviews, of which more will surely need to be made if the ambitions for more deeply historicized and comparative approaches to conjunctural analysis are to be realized in geographical political economy.

One of the interdisciplinary spaces in which geographical political economists can claim a presence is the critical study of neoliberalism. This said, the emergence in recent years of a new genre of critical intellectual histories of neoliberalism has remade this space in ways to which geographers have yet to reciprocate. Slobodian’s *Globalists* illustrates the potential of a creative and expansive mode of inside-out analysis, centered in this case on the transnational constitutionalism of the Geneva School, and bringing to earth (competing) projects of globalization. As Adam Tooze has observed, the book offers a critical counter to the Hayekian conceit of “the economy sublime,” as (if) unknowable, deterritorialized, ungovernable, and effectively beyond politics, pointing to the potentially transcendant role of anti-Hayekian historical geographies:

To move beyond Hayek, what we need to revive is not simply the idea of economic sovereignty, whether on a national or transnational scale, but his true enemies: the impulse to know, the will to intervene, the freedom to choose not privately but as a political body. An anti-Hayekian history … would be one that refuses neoliberalism’s deliberately elevated level of discourse and addresses itself instead to what [the] airy talk of orders and constitutions seeks to obscure: namely, the engines both large and small through which social and economic reality is constantly made and remade, its tools of power and knowledge ranging from cost-of-living indicators to carbon budgets, diesel emission tests and school evaluations. It is here that we meet real, actually existing neoliberalism—and may perhaps hope to counter it (Tooze 2018: 136).

This speaks to a remit that is attentive to dialectical processes of historical change, and to the episodic movement of conjunctural formations, but also to their ongoing coproduction and periodic contestation through localized projects, micropractices, and everyday lifeworlds. The methodological move here is once again a disruptive one: to take on and then push beyond the naturalization of neoliberalism in deregulationist discourse and technocratic normalization.

## IV Conclusion: Worlding ideas

The so-called power of economic ideas, we have argued here, cannot be located on the supply-side alone, in original texts, with influential thinkers, or with intrinsic, essentialized capacities of ideas themselves. To the extent that these powers are realized, they are realized unevenly, in situationally specific ways, and through socially mediated interactions and translations. Since ideas are associated with powers, properties, and potentials that are conjunctural and geohistorically specific, rather than mechanical or universal, there is a distinctive and indeed significant mandate for the development of *geographical* political economies of ideation. To problematize ideation as a sociospatial process is consequently to invite inquiries that are necessarily and reflexively context-specific, rather than just incidentally so. Even as they sometimes take on lives of their own, ideas do not speak for themselves. They do not arrive on the scene as self-propelling bolts from the blue. They are coproduced with the institutionally contoured and unevenly developed worlds in which they intervene, and where they are mobilized to explain, narrate, and in some cases transform. They find meaning, gain traction, and acquire salience—where they do—under certain sociospatial conditions (for instance, in moments or sites of crisis), which is to say that they do so in dialogue, through contestation, and in contexts that are geohistorically contingent.

This means that the charge to put ideas in their place—to trace their mobilization, meanings, and diverse effects in grounded and conjunctural ways—is hardly a secondary one, nor merely a supplement to ideas-school approaches. It calls for critical explorations of ideation as a worldly social process, shaped in conjunction with the moving terrains across which geographical political economy is practiced. Departing from a sympathetic engagement with the ideas school, this paper has sought to make a space for geographical political economies of ideation, in conversation with parallel currents in sociology, history, and heterodox political economy. It is perhaps surprising, given the eclectic disposition and ecumenical habits of contemporary economic geography, that there has not been more engagement with economic ideas, the ideas school or the wider research program of historical institutionalism. In opening this dialogue, our intention is not to advocate a wholesale importation of ideas-school perspectives and problematics. Instead, we have sought in a selective way to draw out lessons, insights, and implications from what have been extended and fruitful debates around the role of ideas, big and small, in processes of political-economic transformation, extending to the consideration of different varieties of constructivist methodology and interpretation. We have sought to identify connections and congruences between the axiomatic commitments of geographical political economy and interdisciplinary currents in heterodox political economy, recognizing that there are many conversations yet to be had, and roads yet to be taken. Looking forward, there do not seem to be many obstacles, but plenty of openings and opportunities.

For its part, the extant literature on the political economy of ideation displays both interdisciplinary range and methodological sophistication, even if its specifically geographical dimensions are seldom prioritized, lurking as background conditions and subtexts, sometimes being marshalled into national differences or network effects. On the other hand, geographical political economists have attended to these issues in suggestive but mostly case-specific ways, having yet to engage in sustained dialogue across approaches and disciplines. Seeking to move beyond this inchoate condition calls for both intermediate theorizing and methodological experimentation—preferably in dialogue. To this end, we have developed three cross-cutting arguments. First, there is a need to explore, in dialogue, variants of constructivist method and explication that are worldly but also wary, problematizing ideation as a situated social process. Second, economic ideas should be engaged not as free-floating “things,” but as socially shaped carriers or “compounds,” coproduced with political struggles and programs and subject to ongoing theorization in relation to the uneven spatial development of state power, capitalist transformation, and contested social relations. And third, there are compelling reasons to analyze the always-contextual powers of ideation in conjunctural terms, capitalizing on middle-range or mezzanine-level frameworks and formulations that span the agentic and the institutional, the semiotic and the social, text and context.

Critical attention is consequently focused on those sites and situations where economic ideas are taken up, articulated, and mobilized in social practice. For geographical political economists, the resulting methodological mandate is anything but singular, as we have illustrated with reference to three especially effective monographs that address the “placing” of economic ideas in quite different ways. These are just some of the methodological pathways that might be followed, however, preferably in ways that are reflexive and dialogic. Experimentation with varieties of constructivism, for example, could work the spaces between “thicker” treatments that emphasize the relatively independent power of ideas, for instance in the performativity approach, and “thinner” alternatives in which ideas become little more than carriers for structurally embedded interests. Rather than just picking a side or splitting the difference, there is an opportunity to mediate between the two, working with the principles and practices of geographical political economy to weave uneven spatial development, embedded knowledge cultures, and entrenched power asymmetries into the analysis.

These are but some of the ways to explore the worldly geographies of economic ideas. Rather than force a binary choice between the study of ideas from the inside-out or from the outside-in, or between ideas in the abstract and ideas in social context, critical attention should be focused on the mutual interactions between ideas and contexts, their coproduction and articulated relations. Hence the invitation to explore the connections between ideas as concepts and ideas in contexts, between ideas in thought and ideas in practice, between the supposedly esoteric realm of ideation and the social life of ideas, on the street as well as in the corridors of power. The point here is not to drive towards a methodologically correct or complete solution, as if to isolate a source of ultimate determination, but instead to make and contest explanations of the worlding of ideas.

## Notes

1. Connections might also be made to more expansive explorations of geographical thought (see Barnes and Sheppard, 2019; Blunt and Wills, 2000; Boyle et al., 2019; Livingstone, 1992), although this is not our primary focus here.

2. See Massey (2013) and James (2024). For further definitional discussion, see Berman (2013), Ban (2018), and Kamkhaji and Radaelli (2022). On causal stories and policy ideas, see Stone (1989) and Béland and Cox (2010). On formatting, see Mitchell (2005). Blyth argues that ideas have the capacity to serve as “the blueprints behind institutions [as well as] the media out of which institutions are constructed” (Blyth et al., 2008: 13).

3. See Blyth, 1997; Campbell, 1998; Hall, 1993; Sikkink, 1991, and the discussions in Hay (2004), Abdelal et al. (2010), Béland and Cox (2010), Berman (2013), and Schmidt (2008).

4. Benner (2024) likewise prefers an encompassing reading of “ideation” (beyond the dictionary definition, as the *formation* of ideas and concepts), so as to embrace the wider role of ideas as sense-making devices mobilized by agents and registering at the collective level.

5. The functions performed by ideas include “heuristic shortcuts” and “strategic tools,” their form ranging from formal models and folk tales to flexible discourses, and from piercing revelations, grand designs, and revolutionary concepts to “fluffy metanarratives” (Carstensen et al., 2024: 1, 5; Best, 2020; Diessner, 2023; McLennan, 2004).

6. This is offered as a methodological elaboration of Polanyi’s (1944: 200, 142) account of the rise and fall of the liberal creed, involving the (pre)figurative and persistent grip of “ruling ideas,” many of which melded with basic (mis)conceptions of market economics and liberal governance, to the point of constituting “the touchstones of rationality in politics.” As such, Polanyi is considered to be one of the original constructivists (Abdelal, 2009).

7. To explore social ideas in articulation, after Hall, is to explore connections, relations, and combinations that “are not necessarily given in all cases [and places], as a law or a fact of life,” since they possess “particular conditions of existence” and must be “positively sustained by specific processes,” which far from being universal or eternal must be “constantly renewed, [and] which can under some circumstances disappear or be overthrown” (Hall, 1985: 113-114).

8. This was a conjunctural terrain coproduced with a wide range of ideas, ideational projects, and ideational movements, big and small, progressive and reactionary, new and repurposed, including inter alia monetarism, sustainability and resilience, employability, austerity, welfare dependency, (new) new deals, and so forth. More generally, ongoing debates around the form, meaning, and explanatory status of neoliberalism were also creatures of these conditions (see Mulder, 2023; Peck, 2013).

9. Fourcade (2009: 12-13) distinguishes her method, first, from orthodox approaches comparison that fail to recognize that “the ‘variables’ that organize the comparison are highly contextual themselves,” and second, from interpretative case-study approaches that, “by emphasizing the irreducibility of differences, and sometimes the irrelevance of categorization itself [run] the risk of falling into pure relativism.”

10. Significant for Slobodian (2018a: 2) is the fact that the foundational ideas of neoliberalism, Geneva-style “were discussed collectively,” *Globalists* emphasizing *group* dynamics (including dissent) and ideational interaction.

11. An instructive methodological comparison can be drawn with Tsang and Cheung’s (2024: 2, 32) “concentric” deconstruction of Xi Jinping Thought, read as the “software,” or operating system, of the party-state, which takes the form of a *simultaneous* analysis of the production of political discourse and the exercise of social power.

12. Because, ontologically speaking, actually existing neoliberalism can only be found in conjunctural and hybrid formations, never alone (Peck, 2013), it follows that “what might appear as logical contradictions at the level of ideas,” for instance between neoliberalism and US-style neoconservatism, are realized in “partially and unsystematically symbiotic” ways at the level of social formations, concrete institutions or political subjectivities (Brown, 2006: 693).
